# A Novel Clinical Scoring Model for Interventional Therapy in Chronic Total Occlusion of the Coronary Artery

**DOI:** 10.1155/2021/9988943

**Published:** 2021-09-17

**Authors:** Bin Xiao, Lang Hong, Xinyong Cai, Hongmin Zhu, Bin Li, Liang Shao

**Affiliations:** ^1^Jiangxi Provincial People's Hospital Affiliated to Nanchang University, No. 92 Aiguo Road, Donghu District, Nanchang 330006, Jiangxi, China; ^2^Department of Cardiology, Beijing Friendship Hospital, Capital Medical University No. 95 Yong An Road, Xuan Wu District, Beijing 100053, China; ^3^Jiangxi Quality Control Center of Coronary Intervention, 2.No. 92 Aiguo Road, Donghu District, Nanchang 330006, Jiangxi, China

## Abstract

**Objective:**

With the rapid development of technology and experience, the current percutaneous coronary intervention of chronic total occlusion (CTO-PCI) preoperative scoring model needs to be updated. This study aimed to evaluate the clinical value of the operator-CTO score in predicting the outcome of interventional therapy for chronic total occlusion of the coronary artery.

**Methods:**

The data of 144 lesions in 130 patients with CTO were analyzed prospectively. The CTO procedures were performed by 10 operators with different skills and experiences. Before the procedures, J-CTO, progress, ORA, recharge, and operator-CTO scores were determined. Then, the clinical, imaging, and procedural data of patients in different operator-CTO score groups and between different operators were compared. The final focus was on comparing the predictive ability of each score on the outcome of CTO-PCI.

**Results:**

The overall technical and procedural success rates were 90.9% and 88.9%, respectively. A decreasing trend in the technical success of CTO-PCI was observed according to the operator-CTO score hierarchy of “easy (≤2 points), moderate (3 points), difficult (4 points), and extremely difficult (≥5 points)” (99.0%, 87.5%, 53.8%, and 25.0%, respectively). All five scoring models were well calibrated, and the area under the curve (AUC) for the operator-CTO score was 0.901 (95% CI: 0.821–0.982, *P* < 0.01), larger than the AUC for the remaining four scoring models, showing excellent ability to predict technical outcomes.

**Conclusion:**

The operator-CTO score is a new clinical scoring tool that can predict the outcome of CTO-PCI and can be used to grade the difficulty of the procedure, with the potential to work well with a broad group of operators.

## 1. Introduction

Coronary artery chronic total occlusion (CTO) is a coronary artery obstructive lesion with a positive TIMI flow grade 0 and an occlusion duration of ≥3 months. It is considered a total occlusive lesion in the case of an ipsilateral bridging collateral or ipsilateral collateral vessel, despite the TIMI flow grade >0 in the occluded distal vessel [1]. Coronary CTOs are relatively common and observed in approximately 15%–25% of patients with coronary artery disease undergoing coronary angiography [2]. Patients with CTO can be relieved by drug therapy alone, but the improvement in long-term cardiac function and survival is not satisfactory [2]. Percutaneous coronary intervention for chronic totally occlusive lesions (CTO-PCI) represents the most challenging and cutting-edge technique in contemporary interventional cardiology. The development of equipment and technology, as well as the increase in operator experience, over the past decade has greatly increased the success rate of CTO-PCI, with multicenter reporting success rates exceeding 80% and some centers achieving rates of more than 90% [1]. Successful revascularization of occluded vessels can effectively relieve angina and improve the patient's cardiac function and clinical outcomes [1, 3, 4]. Also, patients with successful CTO-PCI have a lower risk of death, stroke, and coronary artery bypass grafting compared with those with failed CTO-PCI [5].

Accurate preoperative prediction can better guide the opening of occluded vessels. The preoperative scoring system is a clinical tool that not only quantifies the difficulty of the procedure and reduces the number of interventional strategy conversions, the duration of the procedure, and the risk of related complications but also predicts the likelihood of successful revascularization, which aids in clinical decision making [2, 6]. Some scoring systems are already dedicated to CTOs at present (Table 1). The variables of these scoring systems are based on past CTO databases. They have progressively less predictive power for present-day lesions, and none of them take into account operator factors. Therefore, in this study, the variables of previous CTO scoring models were combined and “operator variables” were included to develop an operator-CTO scoring model. Also, a group of patients undergoing CTO-PCI was prospectively analyzed to elucidate the clinical value of this scoring model for predicting technical outcomes.

## 2. Methods

### 2.1. Study Population

This study included 130 patients with CTO lesions who underwent coronary angiography in the Jiangxi quality control center of coronary intervention between July 2018 and November 2019. Patients were selected based on clinical symptoms and/or evidence of myocardial ischemia and left ventricular function, not on the expected success rate of opening, and on the occurrence or worsening of chest symptoms such as chest tightness and chest pain or coronary angiography-confirmed clinical events beginning with an estimated duration of occlusion greater than 3 months. Patients with a bleeding tendency; those with comorbidities likely to affect procedural outcomes, such as acute cardiogenic shock, acute pericarditis, severe valvular disease, thyroid disease, and infections; and others who were unsuitable for PCI were excluded. All patients signed an informed consent form. The study protocol was approved by the ethics committee and conformed to the Declaration of Helsinki.

The 144 procedures for 130 patients were performed by 10 operators with different skills and experience. The choice of the opening technique and guidewire was entirely at the operator's discretion. Also, 144 operations were completed in the same interventional room. The present study investigated the information of the 10 operators and grouped them according to the time of performing CTO-PCI, the annual number of CTO-PCI performed, and the overall success rate of CTO-PCI in the last year. years: “＜5 years, <60 cases, ＜90% group”as group I (*n* = 2); “<5 years, ≥60 cases, <90% group” as group II (*n* = 1); “≥5 years, <60 cases, <90% group” as group III (*n* = 2); “≥5 years, ≥60 cases, <90% group” as group IV (*n* = 3); and “≥5 years, ≥60 cases, ≥90% group” as group V (*n* = 2).

### 2.2. Definition

CTO: Coronary artery occlusion, positive TIMI blood flow grade 0 and coronary artery occlusion time ≥3 months; if there is ipsilateral bridging collateral or ipsilateral collateral vessel, although the distal vessel TIMI blood flow is occluded, > grade 0 is still regarded as a complete occlusive disease [1].

Time to perform CTO-PCI: the time from the first becoming the main operator of CTO-PCI to this study, in years (years are integers; more than 6 months are counted as 1 year); the time to perform coronary PCI is not included in [7].

Annual CTO-PCI operation volume: the average annual number of CTO-PCI operations obtained by dividing the total CTO-PCI operation volume of the operators by the number of years of execution; the overall success rate of CTO-PCI in the previous year: the number of CTO-PCI technical success cases in the previous year divided by the total number of CTO-PCI cases executed the last year.

Past history of target vessel CABG: surgical coronary artery bypass graft surgery performed at least 3 months before this CTO-PCI operation [8, 9].

Past history of MI: a history of acute myocardial infarction before this CTO-PCI operation.

Blunt occlusion: if the occlusion end does not end in a funnel, triangle, or bead shape, but ends with a knife cut, it is a blunt occlusion [10].

Calcification: as described in the J-CTO score [10], there is little difference between entrance and body and mild and severe calcification; that is, there is calcification in the occluded entrance section or occluded body section.

Angled bend: same as PROGRESS score [11], there are at least 2 bends greater than 70° or 1 greater than 90° in the occluded entrance segment and the occluded body segment.

Occlusion length ≥20 mm: according to EuroCTO Club consensus [2], the occlusion length is selected as 20 mm.

Occlusion of distal vascular disease: same as recharge score [9]; that is, there is significant coronary artery disease after the occlusion segment, and/or the diameter of the distal lumen is less than 2 mm.

The collateral circulation Rentrop grade is less than grade 2: this variable is fuzzy in the PROGRESS score, which is the same as the ORA score [12], namely, grade 0: no collateral circulation; grade 1: collateral vessel filling slowly, vague imaging, and contrast agent. The filling and expelling time are significantly prolonged, and the diameter of collateral vessels is less than 1 mm.

In-stent occlusion: the target vessel has a history of stent implantation, postimaging confirmed that the target vessel is completely occluded, and the time is more than 3 months.

Occlusion: same as the ORA score [12], the occluded segment is located at the opening of the proximal segment of the vessel.

Chronic kidney disease stage 3: chronic kidney structure and dysfunction caused by various reasons for more than 3 months, and glomerular filtration rate (GFR) decreased (<60 ml/min·1.73 m2).

The primary endpoint was technical success, which was defined as the successful revascularization of the CTO vessel, that is, the restoration of the forward blood flow TIMI3, the achievement of <30% stenosis in the stent segment, and no related complications during the operation.

The secondary endpoint was procedural success: technical success and no deaths during hospitalization, myocardial infarction, perforation or bleeding requiring treatment, emergency hemofiltration, and PCI or CABG.

### 2.3. CTO Procedural Strategy

The CTO procedure strategy is mainly determined by the operators. The general principles are as follows: (1) bilateral angiography is used to assess CTO lesions, such as stump condition, tortuosity, and occlusion length. (2) For tapered stumps, the initial strategy is often used, antegrade interventional therapy, including but not limited to Antegrade Wire Escalation (AWE), parallel wire technique, anchoring technique, seesaw technique, side-branch technique, subtimal tracking and rentry, and Intravascular Ultrasound-guided True-lumen Seeking and Tracking (IVUS-TST). (3) For not suitable for antegrade therapy, if there are available collateral vessels, direct retrograde interventional therapy can be adopted, including but not limited to kissing guidewire technology and Reverse Controlled Antegrade and Retrograde subintimal Tracking (R-CART). (4) The Antegrade Dissection Re-entry (ADR) strategy was used for the failure of previous antegrade interventional treatment attempts, poor collateral vessel conditions, or previous retrograde interventional treatment failure, and the following cases show that there is no serious diffuse disease in the distal vessels beyond the occlusion segment, the landing zone does not involve larger branch vessels, and the length of the occlusion segment is longer than 20 mm. (5) Under the premise of ensuring coaxiality, a guiding catheter with strong active support is generally used. EBU, XB, and Amplatz guiding catheters are commonly used in the left coronary artery, and Amplatz and XB RCA are generally used in the right coronary artery. (7) The F/8 F guide catheter is used to prepare for the implementation of IVUS real-time guidance; the 8 F guide catheter, SASUKE dual-lumen microcatheter, and Ping-Pang guide catheter technique are generally used when combined with a KDLC dual-lumen microcatheter and IVUS catheter; and 7 F/8 F guide catheters are often used in the operation of ADR instruments [1].

### 2.4. Establishment of an Operator-CTO Scoring Model

The operator-CTO score integrated the previous CTO system score variables. As shown in Table 2, the scoring system included one clinical variable: previous target vessel CABG history; eight angiographic variables: blunt occlusion, calcification, curvature, occlusion length ≥20 mm, distal occlusion, collateral circulation, in-stent occlusion, and initial occlusion; and three operator variables: annual CTO-PCI volume ≥60 cases, CTO-PCI time ≥5 years, and the overall CTO-PCI success rate of the last year ≥90%; the full score was 9. Among these, the difficulty coefficient of ≤2 points was defined as “simple,” the difficulty coefficient of 3 points was defined as “medium,” the difficulty coefficient of 4 points was defined as “difficult,” and the difficulty coefficient of ≥5 points was defined as “extremely difficult.”

### 2.5. Imaging Evaluation of CTO Lesions

All cases were carefully reviewed and analyzed by two professional doctors before the procedures to improve the accuracy of the angiographic analysis. At the same time, 30 cases of CTO angiography images were randomly selected and evaluated by the aforementioned operator (intraobserver variability) and another senior interventional physician (interobserver variability) to test the consistency of the scores.

### 2.6. Statistical Analysis

Descriptive statistical methods were used to analyze baseline, angiography, and operator-related data. Continuous numerical variables were expressed as mean ± s (normal-distribution data) and median or interquartile distance (nonnormal-distribution data), and categorical variables were expressed as frequency or percentage. The kappa test was used to compare the score consistency between observers. The *t* test or rank-sum test, chi-square test, or Fisher exact probability method was used to compare the mean of two samples. Univariate analysis of variance, chi-square analysis of row and column data, or rank-sum test was also used. The calibration of each scoring model (J-CTO, Cl, recharge, ORA, and new score) was evaluated using the Hosmer–Lemeshow goodness-of-fit test. Finally, the receiver operating characteristic curve (ROC) and the area under the curve (AUC) of each scoring model were calculated to compare the ability of each scoring model to distinguish the technical success.

All statistical data were analyzed using SPSS 22.0 and GraphPad Prism 7.0 data and graphics processing software. A *P* value <0.05 indicated a statistically significant difference.

## 3. Results

### 3.1. Study Population Characteristics

The study population consisted of 130 patients. Furthermore, 144 cases of CTO-PCI were examined by 10 operators with different experiences and techniques. The preoperative images of all lesions were analyzed and scored by two professional doctors; the score between different observers had excellent repeatability (kappa value 0.808). IABP was performed in 10 patients, pericardiocentesis was performed in 3 patients, and sudden cardiac death occurred in 1 patient. In 144 cases of CTO-PCI, 131 cases were technically successful, and the success rate was 91.0%; 128 cases were procedurally successful, the success rate was 88.9%, and the operation strategy conversion was 33.3%. The clinical, angiographic, and procedural features are shown in Tables 3, 4, and 5, respectively. The average age was 62.81 ± 11.27 years; patients were mainly male (80%), with BMI 24.36 ± 3.13 kg/m^2^, hypertension (71.5%), hyperlipidemia (55.4%), smoking history (46.9%), impaired glucose tolerance (36.9%), previous PCI history (50%), previous MI (30.8%), and previous CABG history (0.7%). The most common admission symptom was unstable angina pectoris (77.7%). As shown in Table 4, most patients were right coronary dominant type (68.5%). The most common occlusion vessel was the right coronary artery (47.2%), followed by anterior descending artery (42.2%), circumflex artery (9.7%), and left main artery (0.7%). Among CTO lesions, 57.6% had a blunt occlusion, 91.0% had calcification, 33.3% had a curvature, 88.2% had occlusion length ≥20 mm, 54.2% had distal vascular lesions, 47.9% had an open occlusion, 13.9% had an in-stent occlusion, and 31.9% had a collateral circulation Rentrop grade <2. The average J-CTO score was 3.10 ± 0.96, the average progress-CTO score was 1.32 ± 0.95, the average ORA score was 1.31 ± 1.09, the average recharge score was 3.24 ± 1.02, and the average operator-CTO score was 1.78 ± 1.46. Most of the CTO procedures were performed by forward techniques (51.4% by the single-guidewire technique, 9.7% by the double-guidewire technique, and 4.2% by the ADR technique) and a few by retrograde techniques (25.7%), of which 70.3% were performed via the septal collateral pathway and 25.0% were performed by intravascular ultrasound (IVUS) guidance or exploration. The average number of stents was 2.96 ± 1.28, and the average length of stents was 87.50 ± 39.14 mm. The average fluoroscopy time was 67.56 ± 17.92 min, and the average dosage of the contrast medium was 179.50 ± 44.08 mL.

### 3.2. Comparison of Different Operator-CTO Score Groups

As shown in Table 6, most of the lesions in this study were classified as the “simple group” by the operator-CTO score, with 103 cases and only 4 cases in the extremely difficult group. No significant difference was found in the clinical baseline data between different scoring groups (*P* > 0.05). Among the angiographic characteristics of lesions, the proportion of “collateral circulation Rentrop grade <2, blunt occlusion, curvature, distal vascular disease, and ostial occlusion” increased with the increase in the operator-CTO score; except for “calcification, occlusion length ≥20 mm, and ostial occlusion,” the other variables were higher in different scoring groups. Significant differences were observed between the two groups (*P* < 0.05). The use of antegrade technology and the success rate of opening gradually decreased with the increase in the score, with significant differences (*P* < 0.05). From the “simple group” to the “difficult group,” the proportion and value of operation strategy conversion, 7F guide catheter, x-ray fluoroscopy time, and contrast agent dosage gradually increased, with significant differences between the two groups (*P* < 0.05).

### 3.3. Comparison of CTO-PCI Operators with Different Technologies and Experiences

No significant difference was found in the clinical baseline data between different operator groups (*P* > 0.05). Among the pathological characteristics, significant differences were observed in “blunt occlusion and curvature” in each group (*P* < 0.05). The average Operator-CTO score gradually decreased, while the recharge score increased with the increase in technology and experience. A significant difference was noted between the two groups (*P* < 0.05). The proportion of retrograde technology use and opening gradually increased with the increase in technology and experience, with a significant difference between the three groups (*P* < 0.05). The IVUS use rate and the proportion of intervention strategy conversion were higher in the five groups. X-ray fluoroscopy time and contrast agent dosage gradually decreased with the increase in technology and experience, and a significant difference was found in x-ray fluoroscopy time (*P* < 0.05). Significant differences were observed in the technical and procedural success rates among the groups (*P* < 0.05), as shown in Table 7.

### 3.4. Comparison of Different Scoring Models in Predicting the Outcome of CTO-PCI

**Figure 1:**
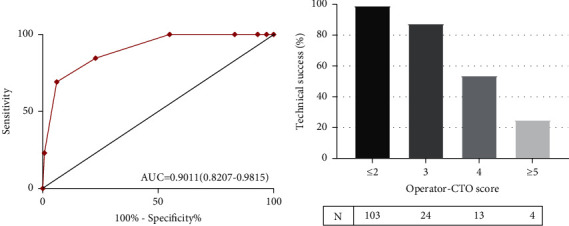
(a) Operator work curve. AUC = 0.9011 (95%CI: 0.821−0.982, *P* < 0.01). (b) The relationship between operator-CTO score and technical success.

**Figure 2:**
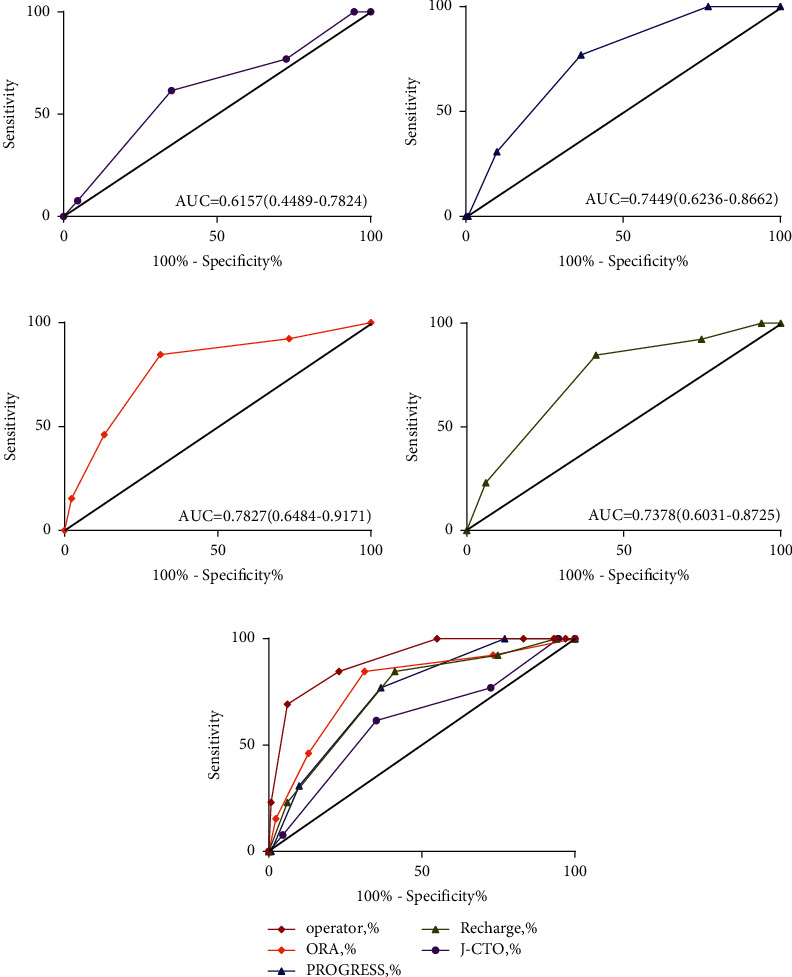
(a) J-CTO score predicts the curve of technical success. (b) Progress-CTO score predicts the curve of ROC. (c) ORA score predicts the curve of ROC. (d) Recharge score predicts the curve of technical success. (e) The ROC curve of the operator score was compared with that of each scoring system.

Hosmer–Lemeshow goodness of fit was used to test the calibration ability of each scoring model: J-CTO score, Hosmer–Lemeshow test (HL) 2 = 2.890, *P*=0.409; progress-CTO score (HL) 2 = 1.013, *P*=0.603; ORA score (HL) 2 = 2.416, *P*=0.299; refresh score (HL) 2 = 1.599, *P*=0.660; and operator-CTO score (HL) 2 = 1.169, *P*=0.883. The five scoring models showed good calibration ability. The relationship between operator-CTO score grouping and technical success probability is shown in Figure 1. The corresponding technical success probability of the simple group (score ≤2), medium group (score 3), difficult group (score 4), and extremely difficult group (score ≥5)” was 99.0%, 87.5%, 53.8%, and 25.0%, respectively. Excellent discrimination ability was observed in ROC analysis.  Figure 2 shows the ROC and AUC: J-CTO score AUC = 0.616 (95% CI: 0.449–0.782, *P* > 0.05); progress-CTO score AUC = 0.745 (95% CI: 0.624–0.866, *P* < 0.01); ORA score AUC = 0.783 (95% CI: 0.648–0.917, *P* < 0.01); and recharge score AUC = 0.738 (95% CI: 0.603–0.873, *P* < 0.01). The AUC of operator-CTO score was larger than that of other scoring models, showing a better ability to predict technical results.

## 4. Discussion

By analyzing the clinical, angiographic, and operator characteristics of each lesion, the score was assigned according to the operator-CTO score model in this study. The score size corresponding to the difficulty of CTO-PCI success (≤2 in the “simple group,” 3 points in the“ medium group,” 4 in the “difficult group,” and ≥5 in the “extremely difficult group”) was 99.0%, 87.5%, 53.8%, and 25.0%, respectively. The operator-CTO score had good calibration ability and better discrimination ability for technical results compared with the previous J-CTO, progress, ORA, and recharge scores.

A comparison of different score groups also revealed that the proportion of most lesion characteristic variables increased with the increase in the score, with differences among the groups, but “calcification, occlusion length ≥20 mm” had no such characteristics, implying that the correlation between different variables and outcomes was different. The clinical and angiographic characteristic variables of the operator-CTO scoring model in this study were obtained by evaluating and integrating the previous scoring model. The previous scoring model was based on the CTO patient database registered in different regions and different periods. The variables with higher correlation with the outcomes were obtained through univariate and multivariate regression analyses, and hence, the model was established. The final influencing variables were different in different periods or different regions. In addition, some variables in the previous scoring systems (Table 1) were greatly affected by the subjective and technical differences in the operator; still, disputes, such as “fuzzy proximal fibrous cap, treatable collateral, and circumflex CTO lesions,” existed [9]. In recent studies, when the most difficult vessel was opened, the variable of progress score “circumflex CTO lesion” showed that the success rate of the left circumflex artery (LCX) was similar to that of the right coronary artery (RCA) and anterior descending artery (LAD) (RCA: 84.5%, LAD: 81.9%, and LCX: 89.2%), which benefited from the progress of technology and the operator's experience [13]. The progress score system was related to CTO lesions. According to the J-CTO score of “first attempt failure,” it was really difficult to find the true cavity in the hematoma or false cavity formed after forward operation, but it was much easier in the center where retrograde or hybrid technology was developed. The variable of “age ≥75 years” was included in the ORA score. The elderly age corresponded to the duration of CTO lesions. The longer the duration of CTO lesions, the more complex the vascular conditions of patients and the lower the success rate of CTO-PCI [14]. However, some studies believed that the duration of CTO was not an important factor for the success of CTO-PCI [15]. Therefore, the aforementioned variables were not included in the scoring model. In clinical practice, many CTO lesions with J-CTO >3 or in extremely difficult groups in other scoring systems could be successfully opened by experienced operators, thus greatly reducing the predictive ability and clinical significance of current scoring systems. The CTO-PCI success rate in all PCI registration studies (54%–80%) was significantly lower than that in experienced centers (85%–90%) [16]. Therefore, with the improvement in technical experience and the continuous updating of equipment, the current scoring system should also be updated, and the variable “operator technology and experience” should be included.

Defining an “experienced operator” is difficult due to the different learning ability and effort level of each operator. A few studies have specifically defined the operator's technical experience and clarified the relationship between it and CTO-PCI results [7, 17, 18]. The operator-CTO scoring model was the first to quantify the operator's experience and be applied to clinical practice. An obvious learning curve exists in the interventional treatment of CTO; the success rate of CTO lesions and CTO procedure complications change in an overall linear manner with the increase in the number of operations [19]. In 2009, Thompson et al. described the 3-year learning curve of 12 operators with different experiences. The total number of CTO-PCI operations was more than 75, with more than 20 retrograde attempts and about 60 operators every year. After 2 years, the success rate of CTO-PCI greatly improved, reaching 90% in the third year [17]. In a study in 2016, a novice operator was observed for 5 years; when the retrograde utilization rate was only 18%, the operation volume of 80 cases/year was maintained, and the final success rate reached 90% [20]. A similar “inflection point” was found in a recent study. The novice CTO operator maintained the operation volume of more than 50 cases/year, and the success rate could approach 90% [19] after 5 years. These studies showed that “annual CTO-PCI operation volume” and “CTO-PCI operation years” were important factors to evaluate the technical experience of operators. The present study did not include the variable of “ retrograde technology” because parallel-guidewire technology, subintimal technology, and IVUS-guided technology were widely used. At present, retrograde technology is recommended only in the antegrade technology, which is difficult to open and has a good side effect. The total success rate of CTO-PCI can be greatly improved with the improvement in the success rate of antegrade technology [20]. Finally, based on previous findings, the consensus of the European CTO club, and the situation of the center, the implementation period of ≥5 years and the annual operation volume of ≥60 cases were selected as the threshold of operator experience division, and the success rate of ≥90% was used to divide “experts” and other operations. It is necessary to divide the technical experience of operators so as to make the right CTO lesions meet the right operators. In the primary stage, it is appropriate to transport patients with complex lesions to more experienced centers or operators in time. The operator-CTO score can help deal with this problem quantitatively and answer the question of whether percutaneous vascular reconstruction is worth it. Moreover, the higher the operator-CTO score, the more the x-ray fluoroscopy time, contrast dose, and frequent instrument operation needed for the opening of the lesion, indicating the increased probability of complications. The division of experience threshold is also helpful to the operator's stage learning, which can ensure the safety to the maximum and gradually improve the success rate.

This study also had some limitations. The sample size of this study was insufficient. Only four lesions were found in the “extremely difficult group (≥5 points),” which could not well reflect the impact of the score stratification change, but reflected the real CTO incidence rate and distribution. Also, the operator-CTO score variable was not established through the standard score card process, but it evaluated and selected the variables of the previous score model and was established according to the clinical practice; its variables need to be verified and optimized in the follow-up study. Finally, the operator-CTO score quantified the operator's experience from “annual operation volume, operation years, and last year's success rate” for the first time; however, the operator, as a complex factor, needs to be further divided by other indicators.

However, the operator-CTO scoring model had a remarkable ability to distinguish the technical success of CTO-PCI, thus helping in clinical decision making and patient selection. It also divided the operator experience threshold, thus helping operators gradually establish a set of standard CTO-PCI learning plans, and had a good clinical value.

## Figures and Tables

**Table 1:** CTO-PCI scoring system based on clinic and angiography.

Variables	Angiographic features						Clinical features					Main disadvantages
	Blunt stump	Lesion length＞20 mm	Calcification	Tortuosity^1^	Collateral^2^	LCx-CTO^3^	Ostial location	Distal lesion	Age (≥75)	Previous reconstruction failure^4^	Previous MI	
J-CTO score	+	+	+	+						+		Not applicable to retrograde or hybrid technology

PROGRESS score	+			+	+	+						Variables are controversial, and the observation of lesions is ignored

Recharge score	+	+	+	+				+		+		Variables related to operator skill or experience were not included

CL score	+	+(1.5 point)	+(2.0 point)			+				+(1.5 point)	+	Not applicable to retrograde or hybrid technology centers

ORA score					+		+		+			Concluded by one operator, not considering the operator's technology and experience

Except for the special marks, the rest is 1 point. ^1^In J-CTO and recharge, the angle was more than or equal to 45°, and it was moderate or severe tortuosity in PROGRESS; ^2^in PROGRESS, it means lack of collateral for treatment, and in ORA, it means collateral Rentrop grade <2; ^**3**^CTO of the circumflex branch in PROGRESS and CTO of non-LAD in CL; ^4^previously failed lesion in J-CTO, and in recharge and Cl, it means previous CABG on the target vessel.

**Table 2:** Operator-CTO score.

Clinical variable			
Previous target vessel CABG history			1 ◻
Angiographic variables			
Blunt occlusion			1 ◻
Calcification			1 ◻
Curvature			1 ◻
Occlusion length ≥20 mm			1 ◻
Distal occlusion			1 ◻
Collateral circulation Rentrop score＜2			1 ◻
In-stent occlusion			1 ◻
Initial occlusion			1 ◻
Operator variables			
Annual CTO-PCI volume ≥60 cases			−1 ◻
CTO-PCI time ≥5 years			−1 ◻
The overall CTO-PCI success rate of the last year ≥90%			−2 ◻
Total			_________
◻simple(≤2)	◻ medium(3)	◻ difficult(4)	◻ extremely difficult(≥5)

**Table 3:** Clinical baseline data of the study population.

Clinical baseline data	(*N* = 130)
Age (year)	62.81 ± 11.27
Male	104 (80%)
BMI (kg/m^2^)	24.36 ± 3.13
Hypertension	93 (71.5%)
Hyperlipidemia	72 (55.4%)
Impaired glucose tolerance	48 (36.9%)
Smoking	61 (46.9%)
Previous PCI history	65 (50%)
Previous CABG history	1 (0.7%)
Previous MI history	40 (30.8%)
≥NYHA III	44 (33.8%)
≥CKD III	13 (10%)
≥2 major coronary artery lesions	123 (94.6%)
Clinic symptoms	
Stable angina	14 (10.8%)
Unstable angina pectoris	101 (77.7%)
Myocardial infarction	15 (11.5%)
LVEF	
≥50%	83 (63.9%)
40%∼50%	35 (26.9%)
＜40%	12 (9.2%)

**Table 4:** Angiographic features of the study population.

Angiographic features	(*N* = 144)
CTO target vessel	
LAD	61 (42.4%)
LCX	14 (9.7%)
RCA	68(47.2%)
LM	1 (0.7%)
Superior vessels	
Right coronary dominance	89 (68.5%)
Left coronary dominance	16 (12.3%)
Balanced type	25 (19.2%)
Collateral circulation, Rentrop＜2	46 (31.9%)
Blunt occlusion	83 (57.6%)
Calcification	48 (33.3%)
Curvature	131 (91.0%)
Occlusion length ≥20 mm	127 (88.2%)
Distal occlusion	78 (54.2%)
Initial occlusion	69 (47.9%)
In-stent occlusion	20 (13.9%)
J-CTO score	3.10 ± 0.96
PROGRESS-CTO score	1.32 ± 0.95
ORA score	1.31 ± 1.09
Recharge score	3.24 ± 1.02
Operator-CTO score	1.78 ± 1.46

**Table 5:** PCI features of the study population.

PCI features	(*N* = 144)
Forward single-guidewire technology	74 (51.4%)
Forward double-guidewire technology	14 (9.7%)
Technology of forward false cavity returning to the true cavity	6 (4.2%)
Retrograde technology	37 (25.7%)
Septal branch channel	26 (70.3%)
Intervention strategy conversion	48 (33.3%)
Intravascular ultrasound guidance/exploration	36 (25.0%)
IABP during the operation	10 (6.9%)
Pericardiocentesis	3 (2.1%)
Postoperative sudden cardiac death	1 (0.7%)
6F guide tube	55 (38.2%)
7F guide tube	89 (61.8%)
Number of stents implanted during operation	2.96 ± 1.28
Total length of the stent implanted during operation	87.50 ± 39.14
X-ray fluoroscopy time	67.56 ± 17.92
Dosage of the contrast agent	179.50 ± 44.08
CTO-PCI technical success	131 (90.9%)
CTO-PCI procedural success	128 (88.9%)

**Table 6:** Comparison of data between different operator-CTO score groups.

	Simple (≤2分)	Medium (3分)	Difficult (4分)	Extremely difficult (≥5)	F*/χ*^2^	*P*
CTO-PCI number	*N* = 103	*N* = 24	*N* = 13	*N* = 4		
Age (years)	62.95 ± 10.95	61.54 ± 11.79	57.69 ± 14.27	63.25 ± 12.15	0.85	0.47
Male	75 (72.8%)	22 (91.7%)	12 (92.3%)	4 (100%)	5.96	0.10
BMI (kg/m^2^)	24.17 ± 3.22	24.31 ± 2.98	24.68 ± 3.72	25.15 ± 2.89	0.91	0.44
Hypertension	71 (68.9%)	16 (66.7%)	8 (61.5%)	3 (75.0%)	0.15	0.93
Hyperlipidemia	51 (49.5%)	17 (70.8%)	8 (61.5%)	3 (75.0%)	4.33	0.12
Impaired glucose tolerance	36 (35.0%)	8 (33.3%)	4 (30.8%)	2 (50.0%)	0.03	0.99
Smoking	45 (43.7%)	11 (45.8%)	10 (76.9%)	1 (25.0%)	2.59	0.27
Previous PCI history	48 (46.6%)	11 (45.8%)	3 (23.1%)	1 (25.0%)	3.40	0.18
Previous CABG history	0	0	1 (7.7%)	0	4.33	0.12
Previous MI history	33 (32.0%)	8 (33.3%)	3 (23.1%)	0	0.50	0.78
≥NYHA III	34 (33.0%)	8 (33.3%)	5 (38.5%)	1 (25.0%)	0.03	0.98
≥CKD III	11 (10.7%)	1 (4.2%)	0	2 (50.0%)	5.21	0.07
Acute coronary syndrome	93 (90.3%)	21 (87.5%)	12 (92.3%)	4 (100%)	0.51	0.82
LVEF＜40%	11 (10.7%)	1 (4.2%)	0	1 (25.0%)	1.40	0.49
Angiographic features						
CTO target vessel					16.56	＜0.05
LAD	48 (46.6%)	5 (20.8%)	7 (53.8%)	1 (25.0%)		
LCX	8 (7.8%)	6 (25.0%)	0	0		
RCA	47 (45.6%)	12 (50.0%)	6 (46.2%)	3 (75.0%)		
LM	0	1	0	0		
Collateral circulation, Rentrop, ＜2	25 (24.3%)	10 (41.7%)	7 (53.8%)	4 (100%)	12.23	＜0.01
Blunt occlusion	48 (46.6%)	18 (75.0%)	13 (100%)	4 (100%)	26.95	＜0.01
Calcification	28 (27.2%)	12 (50.0%)	7 (53.8%)	1 (25.0%)	6.19	＜0.05
Curvature	93 (90.3%)	23 (95.8%)	11 (84.6%)	4 (100%)	1.05	0.59
Occlusion length ≥20 mm	92 (89.3%)	20 (83.3%)	11 (84.6%)	4 (100%)	0.62	0.73
Distal occlusion	50 (48.5%)	14 (58.3%)	10 (76.9%)	4 (100%)	6.92	＜0.05
Initial occlusion	44 (42.7%)	13 (54.2%)	9 (69.2%)	3 (75.0%)	5.08	0.08
In-stent occlusion	11 (10.7%)	8 (33.3%)	1 (7.7%)	0	7.90	＜0.05
PCI features						
Forward single-guidewire technology	76 (73.8%)	13 (54.2%)	4 (30.8%)	1 (25.0%)	13.76	＜0.01
Forward double-guidewire technology	26 (25.2%)	8 (33.3%)	3 (23.1%)	0	1.71	0.66
Intravascular ultrasound guidance/exploration	29 (28.2%)	3 (12.5%)	4 (30.8%)	0	2.87	0.24
Intervention strategy conversion	30 (29.1%)	10 (41.7%)	7 (53.8%)	1 (25.0%)	4.19	0.23
7F guide tube	57 (55.3%)	18 (75%)	12 (92.3%)	2 (50.0%)	7.08	＜0.05
X-ray fluoroscopy time	62.34 ± 16.03	83.32 ± 12.07	90.86 ± 10.25	86	2476.11	＜0.01
Dosage of the contrast agent	166.88 ± 39.30	213.18 ± 25.71	251.43 ± 31.85	210	2782.87	＜0.01
CTO-PCI technical success	99.0%	87.5%	53.8%	25.0%	173.10	＜0.01
CTO-PCI procedural success	96.1%	87.5%	53.8%	25.0%	154.69	＜0.01

**Table 7:** Comparison of different CTO operators.

	I group (<5, <60)	II group (<5, ≥60)	III group (≥5, <60)	IV group (<90%)	V group (≥90%)	F/*χ*^2^	*P*
CTO-PCI number	*N* = 5	*N* = 6	*N* = 13	*N* = 75	*N* = 45		
Age (years)	67.00 ± 6.78	60.83 ± 9.89	59.62 ± 14.22	62.23 ± 11.72	62.44 ± 10.79	0.41	0.80
Male	3 (60.0%)	3 (50.0%)	12 (92.3%)	57 (76.0%)	41 (91.1%)	5.22	0.07
BMI (kg/m^2^)	24.78 ± 2.67	23.61 ± 2.92	24.43 ± 3.62	25.31 ± 2.21	25.09 ± 2.53	1.73	0.62
Hypertension	4 (80.0%)	3 (50.0%)	8 (61.5%)	57 (76.0%)	31 (68.9%)	1.85	0.40
Hyperlipidemia	3 (60.0%)	5 (83.3%)	11 (84.6%)	39 (52.0%)	23 (51.1%)	6.16	0.05
Impaired glucose tolerance	2 (40.0%)	0	3 (23.1%)	30 (40.0%)	17 (37.8%)	2.97	0.23
Smoking	0	0	13 (100.0%)	35 (46.7%)	22 (48.9%)	0.41	0.81
Previous PCI history	1 (20.0%)	5 (83.3%)	3 (23.1%)	31 (41.3%)	26 (57.8%)	3.87	0.14
Previous CABG history	0	0	0	1(1.3%)	0	1.31	0.52
Previous MI history	2 (40.0%)	1 (16.7%)	6 (46.2%)	23 (30.7%)	13 (28.9%)	0.57	0.75
≥NYHA III	2 (40.0%)	0	6 (46.2%)	26 (34.7%)	17 (37.8%)	0.17	0.92
≥CKD III	1 (20.0%)	1 (16.7%)	0	5 (6.7%)	7 (15.6%)	2.45	0.29
Acute coronary syndrome	5 (100.0%)	4 (66.7%)	13 (100.0%)	69 (92.0%)	39 (86.7%)	0.93	0.63
LVEF＜40%	0	1 (16.7%)	2 (15.4%)	6 (8.0%)	4 (8.9%)	0.42	0.81
Angiographic features							
CTO target vessel						1.90	0.49
LAD	3 (60.0%)	3 (50.0%)	5 (38.5%)	29 (38.7%)	21 (46.7%)		
LCX	0	0	2 (15.4%)	6 (8.0%)	6 (13.3%)		
RCA	2 (40.0%)	3 (50.0%)	6 (46.2%)	39 (52.0%)	18 (40.0%)		
LM	0	0	0	1 (1.3%)	0		
Collateral circulation, Rentrop＜2	2 (40.0%)	1 (16.7%)	4 (30.8%)	23 (30.7%)	16 (13.3%)	0.41	0.86
Blunt occlusion	3 (60.0%)	3 (50.0%)	7 (53.8%)	36 (48.0%)	34 (75.6%)	8.89	0.01
Calcification	1 (20.0%)	0	4 (30.8%)	25 (33.3%)	27 (60.0%)	12.33	<0.01
Curvature	4 (80.0%)	5 (83.3%)	12 (92.3%)	68 (90.7%)	42 (93.3%)	0.82	0.63
Occlusion length ≥20 mm	3 (60.0%)	6 (100.0%)	12 (92.3%)	66 (90.7%)	40 (88.9%)	0.35	0.98
Distal occlusion	2 (40.0%)	2 (33.3%)	6 (46.2%)	38 (50.7%)	30 (66.7%)	4.71	0.10
Initial occlusion	3 (60.0%)	3 (50.0%)	6 (46.2%)	34 (45.3%)	23 (51.1%)	4.26	0.81
In-stent occlusion	1 (20.0%)	0	0	11 (14.7%)	8 (17.8%)	3.08	0.21
J-CTO score	2.20 ± 0.84	2.33 ± 1.03	3.08 ± 1.19	3.03 ± 0.92	3.42 ± 0.87	3.67	<0.01
PROGRESS score	1.20 ± 0.84	0.67 ± 0.82	1.23 ± 1.09	1.23 ± 0.97	1.60 ± 0.86	1.96	0.10
ORA score	1.40 ± 0.55	1.00 ± 1.55	1.23 ± 0.83	1.27 ± 1.12	1.42 ± 1.10	0.29	0.88
Recharge score	2.60 ± 1.14	2.67 ± 1.21	3.15 ± 0.99	3.12 ± 0.94	3.6 ± 1.03	2.80	<0.05
Operator score	3.80 ± 0.84	2.33 ± 1.86	2.77 ± 1.30	2.03 ± 1.16	0.73 ± 1.32	14.21	<0.01
PCI features							
Forward single-guidewire technology	1 (20.0%)	5 (83.3%)	12 (92.3%)	53 (70.7%)	23 (51.1%)	5.95	0.05
Forward double-guidewire technology	1 (20.0%)	1 (16.7%)	1 (7.7%)	15 (20.0%)	20 (44.4%)	11.51	<0.01
Intravascular ultrasound guidance/exploration	1 (20.0%)	1 (16.7%)	2 (15.4%)	13 (17.3%)	19 (42.2%)	10.36	<0.01
Intervention strategy conversion	1 (20.0%)	0	4 (30.8%)	24 (32.0%)	19 (42.2%)	3.35	0.19
7F guide tube	3 (20.0%)	4 (66.7%)	10 (76.9%)	43 (57.3%)	29 (64.4%)	1.60	0.45
X-ray fluoroscopy time	78.50 ± 27.58	73.80 ± 26.73	78.46 ± 11.91	67.35 ± 17.66	63.37 ± 17.45	10.12	<0.05
Dosage of the contrast agent	215.00 ± 91.92	192.00 ± 55.41	197.69 ± 42.65	178.68 ± 44.25	172.21 ± 40.51	4.53	0.34
CTO-PCI technical success	40.0%	83.3%	100%	90.7%	95.6%	100.25	<0.01
CTO-PCI procedural success	40.0%	83.3%	100%	89.3%	91.1%	141.29	<0.01

## Data Availability

The data used to support the findings of this study are included within the article.
